# The incremental value of a novel immuno-inflammatory index (SIICI) in predicting sepsis after ureteroscopic lithotripsy: development and validation of a nomogram

**DOI:** 10.3389/fcimb.2026.1755312

**Published:** 2026-02-26

**Authors:** Hongmin Zhou, Jun Luo, Shuai Liu, Heng Cao, Xiangcheng Zhan, Xudong Yao, Dujian Li, Tiancheng Xie, Yunfei Xu

**Affiliations:** 1 Department of Urology, Shanghai Tenth People’s Hospital, Tongji University School of Medicine, Shanghai, China; 2 Department of Urology, Shanghai Fourth People’s Hospital Affiliated to Tongji University School of Medicine, Shanghai, China; 3 Department of Urology, The Third the People’s Hospital of Bengbu, Bengbu Medical College, Bengbu, China

**Keywords:** nomogram, predictive model, sepsis, SIICI, ureteroscopic lithotripsy

## Abstract

**Background:**

Sepsis continues to be a life-threatening complication following ureteroscopic lithotripsy (URSL). Available clinical prediction tools tend to be inadequate in their capacity to depict the underlying pathophysiology of sepsis-systemic immune-inflammatory imbalance. It is particularly difficult in patients who lack obvious preoperative microbiological findings. The study aims to evaluate the new Systemic Immune-Inflammatory Complex Index (SIICI) as well as other indices such as (SII, SIRI, PIV) in predicting post-URSL sepsis.

**Methods:**

We performed a single-center retrospective study of 803 patients who underwent URSL. Multivariate logistic regression was used to create a clinical baseline model. To assess the incremental predictive value, each inflammatory index was added separately to the baseline model. The model performance was compared using the area under the ROC curve (AUC), net reclassification improvement (NRI), integrated discrimination improvement (IDI), likelihood ratio test (LRT) and decision curve analysis (DCA).

**Results:**

The “Base + SIICI” model was found to be the most effective among the four indices. It had the highest degree of discrimination (AUC = 0.863, 95% CI: 0.819-0.908), which is a considerable improvement over the baseline model (AUC = 0.807, p<0.001). There were meaningful improvements in reclassification (NRI = 0.133, p=0.001) and discrimination (IDI = 0.058, p=0.002), a significant likelihood ratio test (p<0.001) backed up these findings. The decision curve analysis confirmed that higher net clinical benefit was found at a larger variety of probability thresholds. Notably, the model performed well in individuals with negative preoperative urine cultures (AUC = 0.850). A visual nomogram was developed and validated based on this model, showing good calibration and a bootstrap-corrected AUC of 0.849. An online calculator was also created to facilitate clinical application.

**Conclusion:**

SIICI is a new index that offers high incremental value in predicting sepsis after URSL compared to traditional indices like SII, SIRI and PIV. Nomogram based on SIICI presents a strong and useful instrument of early stratification of risks of development and can assist in making proactive clinical decisions, particularly where standard infection indicators cannot be used.

## Introduction

Sepsis is a life-threatening organ dysfunction that is a result of the dysfunction of the response to infection of the host. It remains a serious and costly complication after surgery, such as minimal-invasive urological operations like ureteroscopic lithotripsy (URSL) (Singer et al., 2016; Bonkat et al., 2019). Even though URSL is regarded as minimally invasive, there are other factors including high-pressure irrigation, manipulation of instruments and the existence of infectious stones that might cause bacteremia or endotoxin release which can eventually result to sepsis (Wagenlehner et al., 2017; Pietropaolo et al., 2021). Early recognition of the high-risk patients is extremely important to timely implementation of intervention strategies and enhancement of treatment results. This mainly relies on the precision of the risk prediction devices (Laih et al., 2022; Dai et al., 2025).

Existing initiatives aim to develop clinical predicting models that would take into account patient demographics, comorbidities, rountine laboratory results and relevant factors during surgery (Hu et al., 2018; Sugizaki et al., 2025). Nevertheless, these classical indicators have a tendency to be not sensitive or specific enough since they cannot fully represent the essence of sepsis pathophysiologic characteristic of systemic immune inflammatory imbalance (Gao et al., 2025; Saavedra-Torres et al., 2025; Valsamaki et al., 2025). Strong and unbalanced immune response, including the activation, migration and functional modifications of neutrophils, lymphocytes, and monocytes and the formation of a cytokine storm characterizes sepsis, along with severe infection (Bonavia et al., 2023; Tian et al., 2025). Thus, theoretically, combined biomarkers that reflect this systemic immune-inflammatory condition directly might offer more predictive value than conventional markers.

Over the past several years, the combined immune-inflammatory markers of standard peripheral blood cell counts have attracted significant attention because of their low cost-effectiveness and simplicity of access, as well as the abundance of pathophysiological information. For instance, the Systemic Immune-Inflammation Index (SII) (Chen et al., 2025; Chung et al., 2025), Systemic Inflammation Response Index (SIRI) (Agar et al., 2025; Li et al., 2025), and Pan-Immune Inflammation Value (PIV) (Liu et al., 2025; Onal Kalkan et al., 2025) are strongly prognostically valuable both in oncology and in infectious diseases. These markers comprehensively reflect the status of the pro-inflammatory state (neutrophil, monocyte), the state of immunosuppression (lymphocyte reduction), and host responses (platelet), and therefore they offer a broad view of the immune-inflammation network. Nevertheless, the predictive performance of these indicators in specific situations following the occurrence of sepsis, especially when combined with existing clinical models, has not yet been systematically compared and verified.

The novel index, Systemic Immune-Inflammatory Complex Index (SIICI), is calculated as (Neutrophil count × Monocyte count × 1000)/(Platelet count × Lymphocyte count) (Wang et al., 2025). The formula purposefully puts myeloid derived inflammatory cells (neutrophils and monocytes) at the numerator, indicating the hyperinflammatory state (Cheng et al., 2025; Duan et al., 2025), and lymphocytes and platelets at the denominator, indicating their function as the immune competencies and thrombotic regulation respectively (Cilloniz et al., 2021). This integrated ratio is hypothesized to capture the concurrent “myeloid overactivation” and “lymphocyte exhaustion/immunoparalysis” that characterize sepsis, while also incorporating the role of platelets in sepsis-associated immunothrombosis. (Venkata et al., 2013). Hence, SIICI might provide a more comprehensive picture of the dysregulated host responses than the indices which reflect some aspects of these pathways only.

Therefore, this study aims to rigorously evaluate the incremental value of four immune-inflammatory parameters (one of them is SIICI) towards improving the baseline clinical model performance in the prediction of sepsis after URSL. Beyond improvements in model discrimination (AUC) (Hanley and McNeil, 1982), we will use net reclassification improvement (NRI), integrated discrimination improvement (Pencina et al., 2008) as well as likelihood ratio tests and decision curve analysis (DCA) (Vickers and Elkin, 2006)to judge both statistical significance and clinical utility. Besides, we pre-specified subgroup analyses according to preoperative urine culture status in order to clarify the applicability of this model to different patient populations defined in regard to microbiological evidence. The purpose of this analysis is to find out if SIICI predictive utility is conditioned by the presence of culturable bacteriuria or, whether it remains strong by capturing the intrinsic nature of the host immune-inflammatory susceptibility, regardless of the absence of conventional infection signs. We predict that SIICI will show the greatest incremental predictive ability. Finally, based on the optimal model, we will develop and validate a visual nomogram (Balachandran et al., 2015) and an online calculator (Ahn et al., 2025), which will be helpful to clinicians as a powerful and user-friendly tool to classify and predict risks of post-URSL sepsis at an early stage.

## Methods and materials

### Study design and population

A single-center, retrospective cohort study was conducted, enrolling patients who underwent ureteroscopic lithotripsy (URSL) at the Department of Urology, Shanghai Tenth People’s Hospital, between January 1, 2023, and June 30, 2025. A total of 803 patients were included in the final analysis, comprising 57 patients diagnosed with postoperative sepsis and 746 patients in the non-sepsis control group. The study protocol was reviewed and approved by the Institutional Ethics Committee of Shanghai Tenth People’s Hospital (Approval No: SHSY-IEC-5.0/24K100/P01). Given the retrospective nature of the study, the requirement for informed consent was waived. This study was a retrospective exploratory analysis of existing clinical data; therefore, no formal *a priori* sample size calculation was performed. Instead, we included all consecutive eligible patients during the study period. The observed incidence of postoperative sepsis in this cohort was 7.1%, which aligns with the clinically recognized low baseline incidence of this complication following URSL.

**Figure 1:**
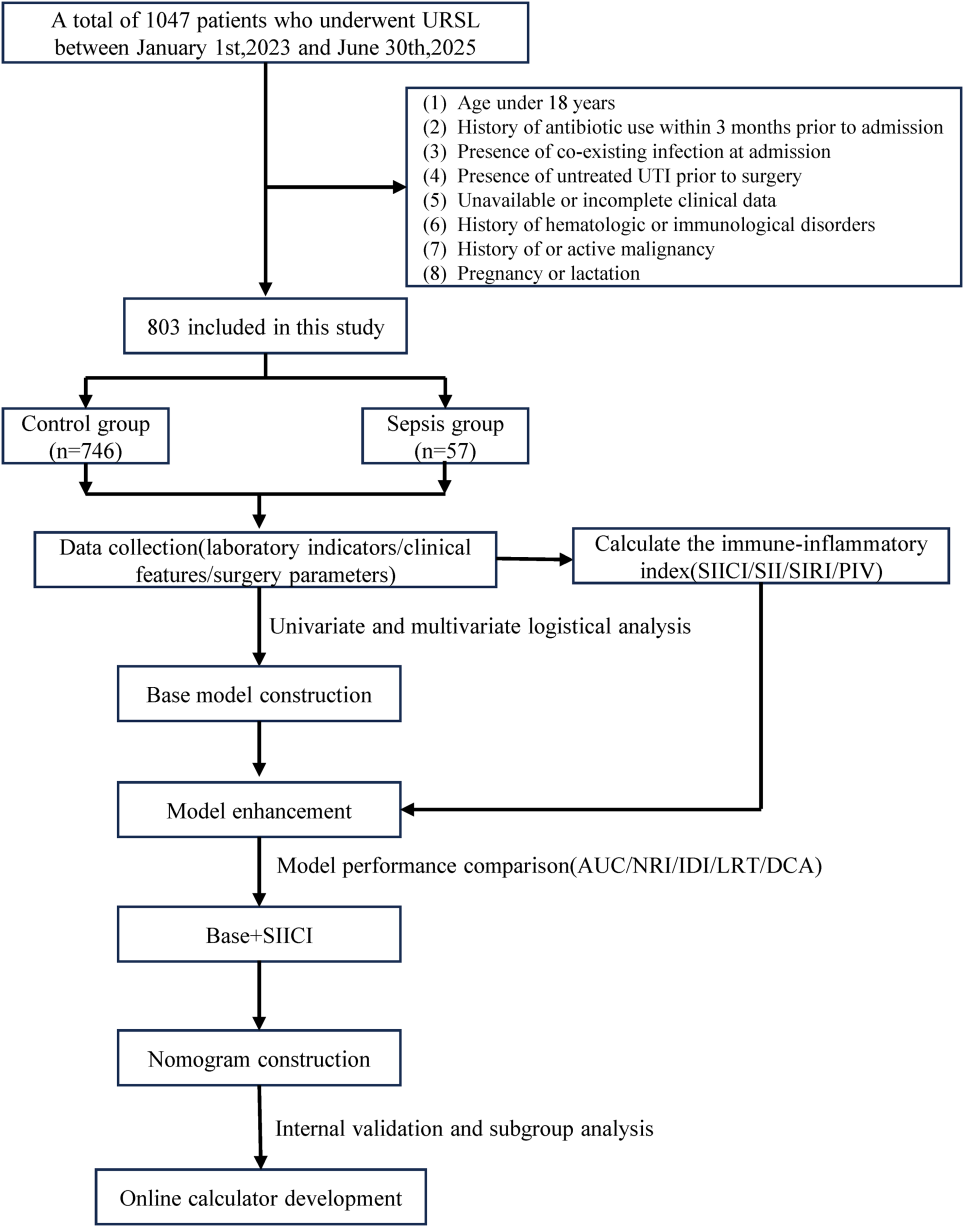
Flow diagram depicting the procedure of subject inclusion.

The inclusion criteria were as follows: (1) age ≥ 18 years; (2) no history of antibiotic use within three months prior to admission; (3) no other known active infections in any organ or tissue before surgery. Exclusion criteria included: (1) untreated urinary tract infection (UTI) or onset of urosepsis prior to surgery; (2) a history of or current diagnosis of malignant tumors; (3) diagnosed hematological diseases, known immune system disorders, or ongoing/prior immunosuppressive therapy; (4) Unavailable or incomplete clinical data; (5) Pregnancy or lactation. A detailed flow diagram of patient screening and inclusion is presented in **Figure 1**.

### Data collection and variable definitions

The primary outcome was the occurrence of postoperative sepsis. Postoperative sepsis is defined according to the international consensus criteria of Sepsis-3, that is, suspected infection accompanied by a quick Sequential Organ Failure Assessment (qSOFA) score of ≥ 2. The selection of the qSOFA score is based on its clinical practicability and rapid assessment ability in the postoperative setting, enabling timely identification of patients with organ dysfunction indicative of sepsis without relying on laboratory-based SOFA scores. Comprehensive data were systematically extracted from electronic medical records, encompassing demographics (age, sex, and body mass index), preoperative laboratory parameters (C-reactive protein [CRP], white blood cell count [WBC], red blood cell count [RBC], hemoglobin [HB], platelet count [PLT], neutrophil count, lymphocyte count, monocyte count, albumin [ALB], creatinine, and uric acid), surgical and stone characteristics (operation time >60 minutes, presence of hydronephrosis, and maximum stone diameter >1.5 cm), and urinalysis and microbiological results (positive urine nitrite, positive urine protein, positive leukocyte esterase, urine WBC count >50/HPF, and positive urine culture). The outliers of laboratory values were handled based on clinical rationality. Furthermore, the following immuno-inflammatory indices were calculated from preoperative complete blood counts (units: 10^9^/L):


$$\begin{array}{l} \text{Systemetic Immune}-\text{Inflamation Index}\left(\text{SII}\right)\\ =\left(\text{Neutrophil count}\times \text{Platelet count}\right)/\text{Lymphocyte count} \end{array}$$

$$\begin{array}{l} \text{Systematic Inflamation Rsponse Index}\left(\text{SIRI}\right)\\ =\left(\text{Neutrophil count}\times \text{Monocyte count}\right)/\text{Lymphocyte count} \end{array}$$

$$\begin{array}{l} \text{Pan}-\text{immune}-\text{inflamation Value}\left(\text{PIV}\right)\\ =(\text{Neutrophil count}\times \text{Platelet count}\\ \times \text{Monocyte count})/\text{Lymphocyte count} \end{array}$$

$$\begin{array}{l} \text{Systematic> Immune}-\text{Inflammatory Complex >Index}\left(\text{SIICI}\right)\\ =\left(\text{Neutrophil count}\times \text{Monocyte count}\times 1000\right)\\ /\left(\text{Platelet count}\times \text{Lymphocyte count}\right) \end{array}$$

While SIICI has been previously described in other clinical contexts (Wang et al., 2025), the present study is the first to evaluate its incremental predictive value specifically for post-URSL sepsis and to compare it directly against SII, SIRI, and PIV within a preoperative risk stratification model.

### Statistical analysis

In light of the class imbalance for the outcome event and the skewed distribution of most laboratory variables, we employed the following analytical strategies to ensure robust results: continuous variables were presented as median (interquartile range) and compared using the Mann-Whitney U test; model performance evaluation primarily relied on the area under the receiver operating characteristic curve (AUC), a metric relatively insensitive to class imbalance, supplemented by reclassification improvement metrics; and internal validation was performed via bootstrap resampling (1000 repetitions) to correct for optimistic bias in performance estimates.

Categorical variables were presented as numbers and percentages and compared using the Chi-square test or Fisher’s exact test, as appropriate. The identification of risk factors was conducted through univariate logistic regression, with variables yielding a p-value < 0.1 subsequently incorporated into a multivariate logistic regression model via a stepwise selection method to establish the base clinical model. Multivariable logistic regression was chosen to construct the baseline clinical model due to its widespread use in clinical prediction, intuitive interpretation via odds ratios, and suitability for assessing incremental value using metrics such as NRI and IDI. To rigorously evaluate the incremental predictive value of each immuno-inflammatory index (SIICI, SII, SIRI, PIV), they were individually added to this base model. The performance of these enhanced models was then comprehensively assessed and compared against the base model by evaluating discrimination through the Area Under the Receiver Operating Characteristic Curve (AUC) with comparisons made using DeLong’s test; measuring reclassification improvement via the Net Reclassification Improvement (NRI) and Integrated Discrimination Improvement (IDI); testing for model fit improvement with the Likelihood Ratio Test (LRT); and analyzing clinical utility across a range of probability thresholds using Decision Curve Analysis (DCA), complemented by Clinical Impact Curves to visualize the net benefit. The model demonstrating the most substantial improvement was selected as the final model and used to construct a visual nomogram for individualized risk prediction. The performance of this final nomogram was subsequently validated by assessing its discrimination (via bootstrap-corrected AUC), calibration (using calibration plots and the Hosmer-Lemeshow test), and clinical utility (DCA). To assess the generalizability and clinical specificity of the final model, a subgroup analysis was performed by applying the ‘Base + SIICI’ nomogram separately to patients with positive and negative preoperative urine cultures. The discriminative performance (AUC) of the model was calculated and compared between these two subgroups. To enhance practical application, a dynamic, web-based version of this nomogram was developed as an online calculator using the DynNom package in R. The developed model is intended for use in adult patients (≥18 years) undergoing elective ureteroscopic lithotripsy, without active preoperative urinary tract infection, immunocompromised status, or malignancy. It is designed as a preoperative risk stratification tool to identify patients at high risk for postoperative sepsis, and its predictions should be interpreted alongside clinical judgment and not as a sole decision-making criterion. All analyses were performed with R software (version 4.0.3), and a two-sided P-value < 0.05 was defined as statistically significant.

## Results

### Study population and baseline characteristics

A total of 803 patients who underwent URSL were included in the final analysis, of whom 57 (7.1%) developed postoperative sepsis. The baseline characteristics are summarized in **Table 1**. Compared to the non-sepsis group, patients who developed sepsis had a significantly lower proportion of males (52.6% vs. 71.8%, p = 0.002) and exhibited a more pronounced inflammatory state. This was evidenced by significantly higher preoperative levels of CRP (25.9 vs. 3.6 mg/L, p < 0.001), WBC (7.7 vs. 6.9 × 10^9^/L, p = 0.001), neutrophils (5.6 vs. 4.5 × 10^9^/L, p < 0.001), and monocytes (0.56 vs. 0.42 × 10^9^/L, p < 0.001), alongside significantly lower levels of lymphocytes (1.28 vs. 1.65 × 10^9^/L, p < 0.001), albumin (41.6 vs. 44.0 g/L, p < 0.001), and platelets (192.0 vs. 232.0 × 10^9^/L, p < 0.001). Several surgical and urinary markers also showed significant differences, including longer operation time (>60 minutes), hydronephrosis, larger stone diameter, positive urine nitrite, positive leukocyte esterase, elevated urine WBC count, and positive urine culture (all p < 0.05). Consequently, all four immuno-inflammatory indices (SIICI, SII, SIRI, PIV) showed highly significant differences between the two groups (all p < 0.001), with the sepsis group demonstrating markedly elevated values.

**Table 1:** Baseline characteristics and preoperative profiles of sepsis and non-sepsis patients undergoing ureteroscopic lithotripsy.

Variables	Sepsis(n=57)	Non-sepsis(n=746)	P value
Age, median (IQR), years	60.0(52.5-64.5)	58.0(46.0-66.0)	0.180
Male,n(%)	30(52.6%)	436(71.8%)	0.002
Hypertension,n(%)	19(33.3%)	227(30.5%)	0.651
Diabetes,n(%)	18(31.6%)	158(21.2%)	0.068
BMI, median (IQR),kg/m^2^	24.2(22.5-26.4))	24.6(22.5-26.9)	0.262
CRP, median (IQR), mg/L	25.9(4.6-46.8)	3.6(1.5-8.8)	<0.001
WBC, median (IQR), 10^9^/L	7.7(6.7-9.3)	6.9(5.8-8.3)	0.001
RBC, median (IQR), 10^12^/L	4.39(4.06-4.80)	4.52(4.19-4.84)	0.160
HB, median (IQR), g/L	133.0(119.5-146.0)	138.0(126.0-147.0)	0.075
PLT, median (IQR), 10^9^/L	192.0(152.0-238.5)	232(197.8-275.0)	<0.001
Neutrophil, median (IQR), 10^9^/L	5.6(4.5-7.2)	4.5(3.5-5.7)	<0.001
Lymphocyte, median (IQR), 10^9^/L	1.28(0.99-1.59)	1.65(1.28-2.06)	<0.001
Monocyte, median (IQR), 10^9^/L	0.56(0.45-0.66)	0.42(0.33-0.52)	<0.001
ALB, median (IQR), g/L	41.6(39.9-43.5)	44.0(41.7-46.0)	<0.001
creatinine, median (IQR), umol/L	89.6(72.8-129.7)	83.5(67.1-113.8)	0.082
uric acid, median (IQR), umol/L	374.0(311.2-422.7)	361.0(294.9-431.7)	0.657
Oeration Time>60min,n(%)	22(38.6%)	169(22.7%)	0.006
Hydronephrosis,n(%)	45(78.9%)	399(53.5%)	<0.001
Stone maximum diameter>1.5cm,n(%)	16(28.1%)	97(13.0%)	0.002
Positive urine nitrite, n(%)	8(14.0%)	19(2.5%)	<0.001
Positive urine protein, n(%)	17(29.8%)	158(21.2%)	0.128
Positive leukocyte esterase,n(%)	39(68.4%)	298(39.9%)	<0.001
Urine WBC count>50,n(%)	23(40.4%)	173(23.2%)	0.004
Positive urine culture,n(%)	22(38.6%)	87(11.7%)	<0.001
SIICI, median (IQR)	12.79(7.77-20.28)	4.77(3.07-7.74)	<0.001
SII, median (IQR),	866.4(619.2-1217.9)	641.9(468.5-923.8)	<0.001
SIRI, median (IQR),	2.27(1.69-3.51)	1.10(0.73-1.77)	<0.001
PIV, median (IQR),	428.1(324.5-671.6)	259.6(165.0-433.9)	<0.001

BMI, Body Mass Index; CRP, C-reactive Protein; WBC, White Blood Cell; RBC, Red Blood Cell; HB, Hemoglobin; PLT, Platelet; ALB, Albumin; SIICI, systemic immune-inflammatory complex index; SII, systemic immune-inflammation index; SIRI, systemic inflammation response index; PIV, pan-immune-inflammation value.

### Predictors of postoperative sepsis and base model construction

Univariate logistic regression identified multiple factors associated with postoperative sepsis (**Table 2**). Multivariate analysis subsequently identified five independent predictors, which collectively constituted the base clinical model: CRP (OR 1.016, 95% CI 1.008-1.025, p < 0.001), albumin (OR 0.910, 95% CI 0.841-0.986, p = 0.021), hydronephrosis (OR 3.892, 95% CI 1.928-7.855, p < 0.001), positive leukocyte esterase (OR 2.250, 95% CI 1.199-4.225, p = 0.012), and positive urine culture (OR 3.259, 95% CI 1.705-6.229, p < 0.001). To avoid collinearity, the immune-inflammatory indicators were incorporated and assessed in the separate statistical models (**Supplementary Table 1**).

**Table 2:** Univariate and multivariate logistic regression analyses of risk factors for postoperative sepsis.

Variables	Univariate logistic analysis			Multivariate logistic analysi		
	OR	95%CI	p	OR	95%CI	p
Age	1.016	0.994-1.038	0.167			
Male	0.435	0.253-0.750	0.003			
BMI	0.955	0.881-1.035	0.267			
Hypertension	1.141	0.644-2.022	0.652			
Diabetes	1.715	0.955-3.079	0.071			
CRP	1.019	1.011-1.027	<0.001	1.016	1.008-1.025	<0.001
WBC	1.170	1.047-1.308	0.006			
RBC	0.734	0.435-1.240	0.248			
HB	0.986	0.972-1.001	0.065			
ALB	0.868	0.809-0.931	<0.001	0.910	0.841-0.986	0.021
Creatinine	1.001	0.999-1.004	0.292			
Uric acid	1.001	0.999-1.003	0.468			
Oeration Time>60min	2.146	1.226-3.758	0.008			
Hydronephrosis	3.261	1.698-6.265	<0.001	3.892	1.928-7.855	<0.001
Stone maximum diameter>1.5cm	2.611	1.410-4.834	0.002			
Positive urine nitrite	6.247	2.604-14.989	<0.001			
Positive urine protein	1.582	0.873-2.865	0.130			
Positive leukocyte esterase	3.257	1.829-5.802	<0.001	2.250	1.199-4.225	0.012
Urine WBC count>50	2.241	1.285-3.906	0.004			
Positive urine culture	4.761	2.671-8.489	<0.001	3.259	1.705-6.229	<0.001

BMI, Body Mass Index; CRP, C-reactive Protein; WBC, White Blood Cell; RBC, Red Blood Cell; HB, Hemoglobin; ALB, Albumin.

### Incremental predictive value of immuno-inflammatory indices

**Figure 2:**
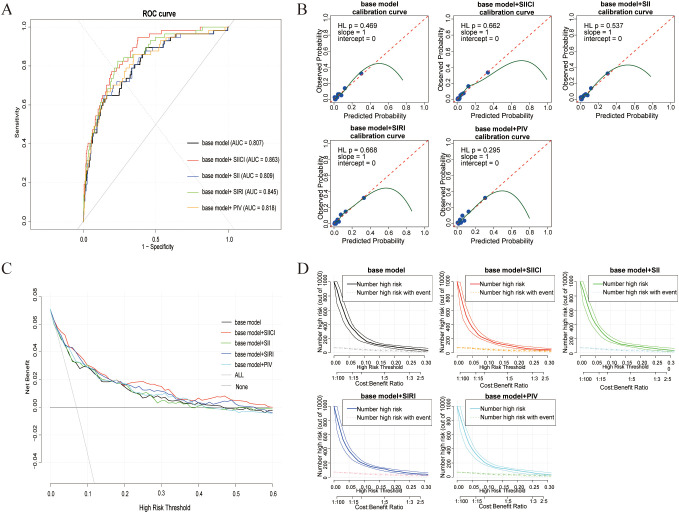
Performance comparison of the base model and models incorporating immuno-inflammatory indicators. **(A)** Receiver operating characteristic (ROC) curves comparing the discrimination ability of the models. **(B)** Calibration curves for the different prediction models. **(C)** Decision curve analysis (DCA) showing the net benefit of different prediction models across various threshold probabilities. **(D)** Clinical impact curve for the different prediction models, depicting the number of high-risk patients identified versus the number of true positives across risk thresholds.

The base model demonstrated good discrimination with an AUC of 0.807 (95% CI: 0.746-0.867). The addition of each immuno-inflammatory index was then rigorously assessed for its incremental value. The model incorporating the SIICI index yielded the highest improvement in discrimination, with an AUC of 0.863 (95% CI: 0.819-0.908, p < 0.001 vs. base model). The addition of SIRI also significantly enhanced the AUC to 0.845 (95% CI: 0.794-0.896, p < 0.001). In contrast, the inclusion of SII (AUC = 0.809, p=0.480) or PIV (AUC = 0.818, p=0.076) did not result in a statistically significant improvement (**Table 3** and **Figure 2A**).

**Table 3:** Assessment of the incremental value of immuno-inflammatory indicators based on AUC.

Models	AUC	95%CI	P value
Base model	0.807	0.746-0.867	Reference
Base model+SIICI	0.863	0.819-0.908	<0.001
Base model +SII	0.809	0.748-0.870	0.480
Base model+SIRI	0.845	0.794-0.896	<0.001
Base model+PIV	0.818	0.759-0.877	0.076

SIICI, systemic immune-inflammatory complex index; SII, systemic immune-inflammation index; SIRI, systemic inflammation response index; PIV, pan-immune-inflammation value.

Superior calibration of the extended models, evidenced by the close alignment of their calibration curves with the ideal line (**Figure 2B**), was further confirmed quantitatively. Specifically, the Base + SIICI model demonstrated excellent calibration with a non-significant Hosmer-Lemeshow test (p = 0.662), ruling out systematic miscalibration.

These findings were corroborated by the Net Reclassification Improvement (NRI) and Integrated Discrimination Improvement (IDI) metrics (**Table 4**). The Base + SIICI model achieved a significant NRI of 0.133 (95% CI: 0.053-0.213, p=0.001) and an IDI of 0.058 (95% CI: 0.022-0.094, p=0.002). The Base + SIRI model showed a significant IDI of 0.034 (p=0.014), though its NRI was not statistically significant (0.021, p=0.435). The models incorporating SII and PIV did not demonstrate significant improvements in either NRI or IDI.

**Table 4:** Assessment of the incremental value of immuno-inflammatory indicators based on reclassification and discrimination (NRI and IDI).

Models	NRI(95%CI)	p	IDI(95%CI)	p
Base model+SIICI	0.133 (0.053-0.213)	0.001	0.058 (0.022-0.094)	0.002
Base model+SII	-0.012 (-0.049-0.024)	0.512	0.002 (-0.004-0.007)	0.542
Base model+SIRI	0.021 (-0.032-0.074)	0.435	0.034 (0.007-0.062)	0.014
Base model+PIV	-0.004(-0.060-0.047)	0.872	0.009 (-0.004-0.021)	0.185

SIICI, systemic immune-inflammatory complex index; SII, systemic immune-inflammation index; SIRI, systemic inflammation response index; PIV, pan-immune-inflammation value.

The Likelihood Ratio Test (LRT) further confirmed that adding SIICI (LRT Statistic=23.50, p<0.001) and SIRI (LRT Statistic=17.64, p<0.001) significantly improved the overall model fit, whereas adding SII did not (**Table 5**).

**Table 5:** Assessment of the incremental value of immuno-inflammatory indicators based on likelihood ratio test.

Models	Likelihood ratio test (LRT) statistic	Df	P-value
Base model+SIICI	23.50	1	<0.001
Base model+SII	1.28	1	0.257
Base model+SIRI	17.64	1	<0.001
Base model+PIV	5.59	1	0.018

SIICI, systemic immune-inflammatory complex index; SII, systemic immune-inflammation index; SIRI, systemic inflammation response index; PIV, pan-immune-inflammation value.

### Subgroup analysis based on preoperative urine culture

To further delineate the clinical utility of the SIICI-enhanced model, we evaluated its performance in subgroups stratified by preoperative urine culture. Remarkably, the model demonstrated robust and superior discriminative ability in the culture-negative subgroup (AUC = 0.850, 95% CI:0.793-0.908), outperforming its performance in the culture-positive subgroup (AUC = 0.807, 95% CI:0.714-0.901). This finding indicates that the predictive power of the model, and by extension the SIICI index, is particularly strong in identifying patients at risk of sepsis even in the absence of traditional microbiological evidence of infection.

### Clinical utility and impact

The clinical value of the novel indices was further underscored by Decision Curve Analysis (DCA), which quantified their superior net benefit over the base model for clinical decision-making (**Figure 2C**). The clinical impact curve visually reinforced this finding, showing a close alignment between the number of patients identified as high-risk by the Base + SIICI model and the actual number of true positive cases across threshold probabilities (**Figure 2D**). This superior utility was quantified, as the model identified an average of 100.69 high-risk patients, including 29.31 true positives, yielding the highest efficiency ratio of 0.291 (**Table 6**). This indicates that the SIICI-enhanced model was the most effective in correctly identifying patients who would truly benefit from preemptive interventions.

**Table 6:** Assessment of the incremental value of immuno-inflammatory indicators based on the clinical impact.

Models	Average high-risk patients	Average true positives	Efficiency ratio
Base model+SIICI	100.69	29.31	0.291
Base model+SII	105.88	26.46	0.250
Base model+SIRI	105.04	28.69	0.273
Base model+PIV	108.19	27.62	0.255
Base model	105.58	26.04	0.247

SIICI, systemic immune-inflammatory complex index; SII, systemic immune-inflammation index; SIRI, systemic inflammation response index; PIV, pan-immune-inflammation value.

### Nomogram development and validation

**Figure 3:**
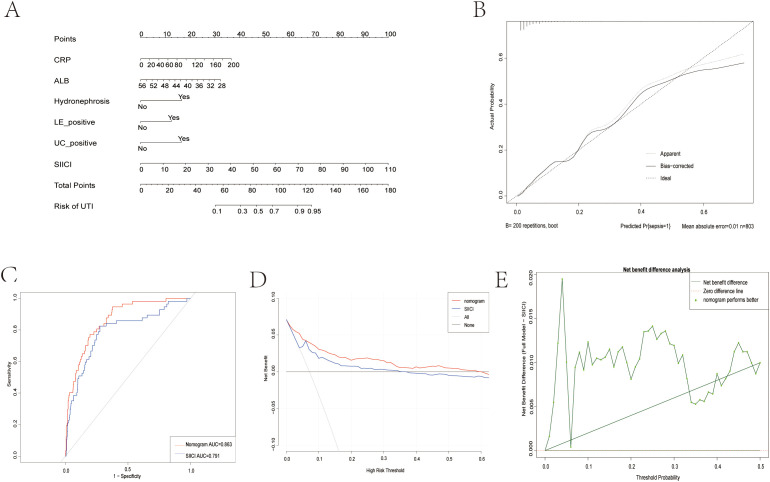
The predictive nomogram and its validation for predicting post-URSL sepsis. **(A)** The developed nomogram based on the Base + SIICI model. **(B)** The ROC curve showing the discriminative performance of the nomogram in the study cohort. **(C)** The calibration curve of the nomogram. **(D)** Decision curve analysis evaluating the clinical utility of the nomogram. **(E)** Analysis of the net benefit difference.

**Figure 4:**
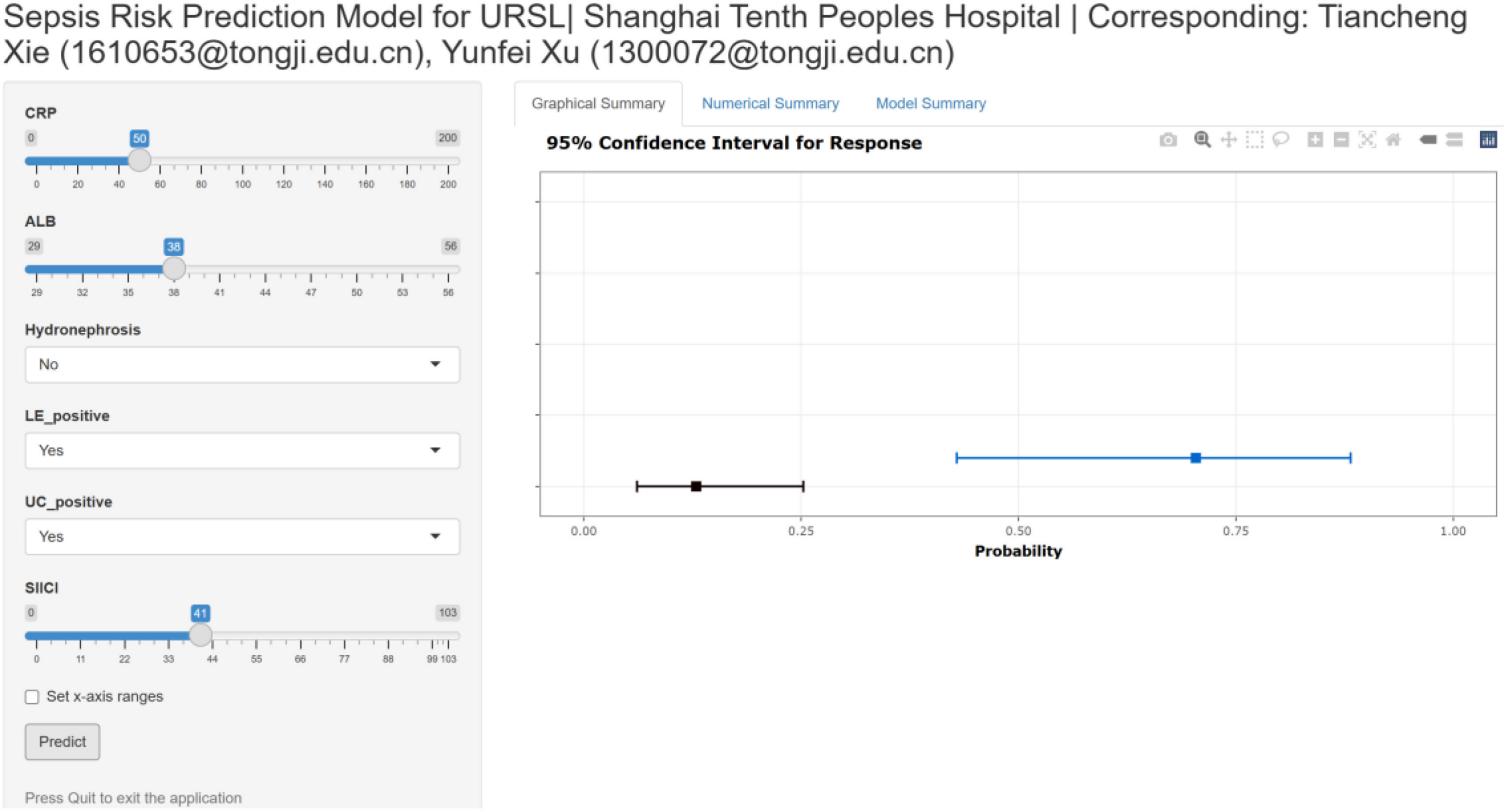
An online calculator converted from the nomogram is available for generating individualized predictions of post-URSL sepsis.

Based on its superior and consistent performance across all metrics, the Base + SIICI model was selected to construct a visual nomogram for the individualized prediction of post-URSL sepsis (**Figure 3A**). The nomogram demonstrated robust performance, with excellent discrimination (AUC = 0.863, bootstrap-corrected AUC = 0.849, **Figure 3B**) and good calibration (**Figure 3C**). Decision curve analysis further confirmed the model’s clinical value, demonstrating superior net benefit across a wide range of clinically relevant threshold probabilities compared to alternative strategies (**Figure 3D**), supported by the analysis of the net benefit difference (**Figure 3E**).To bridge the gap between research and clinical practice, an interactive, web-based calculator derived from this nomogram was developed(https://sepsisriskprediction.shinyapps.io/DynNom/), providing a user-friendly platform for real-time risk assessment at the point of care (**Figure 4**).

## Discussion

In this retrospective cohort study, we systematically developed and validated a preoperative predictive model for sepsis following ureteroscopic lithotripsy (URSL), with a core focus on evaluating the incremental predictive value of a novel systemic immune-inflammatory complex index (SIICI) relative to established immune-inflammatory indices (SII, SIRI, PIV). The key finding of our study is that the integration of SIICI into a baseline clinical model significantly enhanced predictive performance across multiple metrics (discrimination, reclassification, clinical utility), outperforming SII, SIRI, and PIV by a substantial margin. We further developed and validated a visual nomogram and a user-friendly online calculator based on the Base + SIICI model, which exhibits robust calibration and discrimination (AUC = 0.863, bootstrap-corrected AUC = 0.849) and maintains excellent performance even in patients with negative preoperative urine cultures. These findings not only identify SIICI as a clinically valuable biomarker for post-URSL sepsis but also provide a practical, individualized risk stratification tool for urologists, addressing the unmet clinical need for accurate prediction of this life-threatening complication in the context of minimally invasive urological surgery.

A critical mechanistic explanation for the superior performance of SIICI lies in its unique formula design, which is precisely tailored to capture the core pathophysiological characteristics of sepsis: concurrent hyperinflammation and immune suppression, combined with immunothrombosis. Sepsis is defined by a dysregulated host immune response to infection, characterized by the overactivation of myeloid-derived inflammatory cells (neutrophils and monocytes) that drive a pro-inflammatory cytokine storm, alongside the exhaustion of lymphocytes that mediate adaptive immunity and the dysfunction of platelets that regulate thrombosis and immune responses (Gao et al., 2025; Valsamaki et al., 2025). Unlike existing indices, SIICI is calculated as (Neutrophil count × Monocyte count × 1000)/(Platelet count × Lymphocyte count), which intentionally places hyperactivated myeloid cells in the numerator and immune-competent/thromboregulatory cells (lymphocytes, platelets) in the denominator. This design allows SIICI to quantitatively integrate three interrelated pathological processes of sepsis in a single index: myeloid overactivation, lymphocytic exhaustion, and platelet dysfunction (Samuelsen et al., 2024). In contrast, SII only incorporates neutrophils, platelets, and lymphocytes (excluding monocytes, a key mediator of the pro-inflammatory response), SIRI omits platelets (a critical player in sepsis-associated immunothrombosis), and PIV simply multiplies all four cell types without distinguishing their opposing roles in sepsis pathophysiology (Agar et al., 2025; Chung et al., 2025; Onal Kalkan et al., 2025).Our baseline data (**Table 1**) further support this mechanistic rationale: the sepsis group exhibited marked elevations in neutrophils and monocytes, and significant reductions in lymphocytes and platelets-changes that directly amplify the SIICI value and make it a more sensitive and specific reflection of sepsis-related immune-inflammatory dysregulation than the other indices. This mechanistic alignment between SIICI and sepsis pathophysiology is the fundamental reason for its superior incremental predictive value, and it also explains why SIICI can capture subtle immune-inflammatory perturbations that are not detected by single biomarkers or traditional composite indices.

The comprehensive statistical evaluation of SIICI further confirms its robust predictive value, going beyond simple improvements in AUC to demonstrate meaningful clinical utility. The Base + SIICI model achieved a significant NRI (0.133, p=0.001) and IDI (0.058, p=0.002), indicating that SIICI not only enhances the model’s ability to distinguish between sepsis and non-sepsis patients but also improves the accuracy of individual risk stratification—a critical outcome for clinical practice, as it allows urologists to more precisely identify patients who require proactive intervention rather than just distinguishing high- and low-risk groups. The non-significant Hosmer-Lemeshow test (p=0.662) and well-calibrated calibration curve confirm that the model’s predicted risk probabilities closely align with actual sepsis incidence across the entire risk spectrum, eliminating systematic miscalibration that plagues many clinical prediction models and ensuring reliable risk estimates for individual patients. Most importantly, decision curve analysis (DCA) and clinical impact curve analysis demonstrated that the Base + SIICI model provides a higher net clinical benefit across a wide range of probability thresholds and the highest efficiency ratio (0.291) among all models. This means that the SIICI-enhanced model minimizes unnecessary interventions while maximizing the identification of true high-risk patients—a key consideration in clinical practice, where over-stratification leads to avoidable medical costs and patient anxiety, and under-stratification results in missed opportunities for preemptive management of sepsis.

A pivotal and novel finding of our study is the superior discriminative ability of the SIICI-based model in patients with negative preoperative urine cultures (AUC = 0.850), which even exceeds its performance in culture-positive patients (AUC = 0.807). This finding extends the traditional clinical paradigm, which has relied on microbiological evidence (e.g., positive urine culture), by providing an additional means to identify patients at risk of post-URSL sepsis. Conventional urine culture only detects culturable bacteria, and negative results do not rule out the presence of non-culturable pathogens, bacterial biofilms, or endotoxin release—all of which are common in patients with ureteral stones and can trigger sepsis by inducing host immune-inflammatory dysregulation (Dai et al., 2025; Sugizaki et al., 2025). Our results strongly suggest that SIICI is not merely a surrogate for bacterial burden but a sensitive measure of the host’s intrinsic immune-inflammatory susceptibility. In urine culture-negative patients, the risk of post-URSL sepsis is driven not by overt bacterial infection but by pre-existing immune-inflammatory dysregulation that renders the host vulnerable to infection-induced organ dysfunction; SIICI quantifies this dysregulation and thus identifies the “hidden high-risk host” that would otherwise be missed by standard microbiological and clinical assessments. This is a critical clinical advance, as approximately 88.3% of patients in our cohort had negative preoperative urine cultures (**Table 1**)—a proportion consistent with real-world clinical practice—and our model enables targeted risk stratification and proactive management for this large, previously under-recognized high-risk subgroup.

When contextualizing our findings with previous research on post-URSL sepsis prediction, the novelty and advantages of our study become clear. Prior studies have developed prediction models for post-URSL sepsis based on clinical factors, single inflammatory biomarkers, or machine learning algorithms (Hu et al., 2018; Pietropaolo et al., 2021; Dai et al., 2025; Sugizaki et al., 2025). For example, Pietropaolo et al. developed a machine learning model for post-URSL urosepsis requiring ICU admission, but it relied on a multicenter European cohort and lacked validation in Asian populations (Pietropaolo et al., 2021); Hu et al. developed a nomogram for ureteral calculi-associated urosepsis, but it did not incorporate composite immune-inflammatory indices (Hu et al., 2018). In contrast, our study is the first to evaluate the incremental value of SIICI in post-URSL sepsis prediction and to directly compare it with three widely used immune-inflammatory indices (SII, SIRI, PIV) in a single-center Asian cohort with rigorous statistical methods (AUC, NRI, IDI, LRT, DCA). Our SIICI-based nomogram also has the advantage of simplicity and clinical feasibility: all variables in the model (CRP, albumin, hydronephrosis, positive leukocyte esterase, positive urine culture, SIICI) are readily available from routine preoperative clinical and laboratory assessments, without the need for specialized testing or complex machine learning algorithms. The development of an online calculator (https://sepsisriskprediction.shinyapps.io/DynNom/) further bridges the gap between research and clinical practice, allowing urologists to obtain real-time, individualized sepsis risk estimates at the point of care—an essential feature for translating predictive models into daily clinical practice (Ahn et al., 2025).

The clinical translational potential of our SIICI-based nomogram and online calculator is substantial, with direct applications in preoperative risk stratification and personalized patient management for URSL. For urologists, the model allows for quantitative, evidence-based preoperative counseling of patients, enabling clear communication of sepsis risk and shared decision-making regarding surgical planning (e.g., timing of URSL, preoperative prophylactic antibiotics, postoperative monitoring intensity). For high-risk patients identified by the model (e.g., those with high SIICI values, hydronephrosis, and positive leukocyte esterase), proactive interventions such as extended preoperative antibiotic prophylaxis, intraoperative low-pressure irrigation, and postoperative intensive care unit monitoring can be implemented to mitigate sepsis risk—interventions that have been shown to reduce the incidence and severity of post-URSL sepsis (Wagenlehner et al., 2017; Bonkat et al., 2019). For low-risk patients, the model avoids unnecessary interventions and reduces healthcare costs, aligning with the principles of value-based medicine. Beyond URSL, our findings also raise the possibility that SIICI may be a valuable predictive biomarker for sepsis in other surgical and clinical settings (e.g., percutaneous nephrolithotomy, abdominal surgery), where immune-inflammatory dysregulation is a key driver of sepsis; future research can explore this broader applicability.

## Study limitations

Several limitations of our study warrant acknowledgment. First, its single-center, retrospective design inherently carries risks of selection bias and limits the generalizability of our findings. External validation in multi-center, prospective cohorts is essential to confirm the robustness and transportability of our model. Second, while our model demonstrates strong predictive performance, the absolute number of sepsis events was relatively small, which may affect the stability of the estimates. Larger studies are needed to refine the model coefficients. Third, our study is subject to the inherent challenge of class imbalance, with only 57 sepsis events (7.1%) among 803 patients. While this proportion reflects the true, low incidence of post-URSL sepsis in clinical practice, it may affect the precision and stability of the logistic regression coefficients and increase the risk of model overfitting. Although we employed bootstrap validation and imbalance-resistant metrics (AUC, NRI, IDI), future studies with larger, multicenter cohorts or those employing advanced sampling techniques (e.g., oversampling) are warranted to further validate and refine the model. Fourth, our model is based on logistic regression. While this provides high clinical interpretability, we acknowledge that other advanced machine learning algorithms were not compared in this study. Future research could involve head-to-head comparisons between logistic regression and various machine learning models to determine the optimal predictive framework for post-URSL sepsis. Finally, the model is based on preoperative variables; incorporating dynamic postoperative changes in laboratory values or clinical status might further improve predictive accuracy but was beyond the scope of this initial investigation.

## Conclusions

In conclusion, this study identifies the SIICI index as a powerful and clinically valuable biomarker that significantly improves the prediction of sepsis following URSL. By comprehensively capturing the underlying immune-inflammatory dysregulation, SIICI provides incremental value over both a base clinical model and other immuno-inflammatory indices. The validated nomogram and online calculator we have developed offer a practical means to leverage this information, paving the way for improved risk stratification and personalized patient management in urological surgery. Crucially, its exceptional performance in patients with negative urine cultures highlights its unique role in identifying the “hidden high-risk host” based on immune phenotype rather than relying solely on microbiological evidence. Given the retrospective nature of the study, the requirement for informed consent was waived.

## Data Availability

The raw data supporting the conclusions of this article will be made available by the authors, without undue reservation.
